# Supplementary material for: Evaluation of Athlete Monitoring Tools across 10 Weeks of Elite Youth Basketball Training: An Explorative Study
Source: Sports (Basel). 2023 Jan 25;11(2):26. doi: 10.3390/sports11020026 (PMC9967008; doi:10.3390/sports11020026)
Supplement: Supplementary file 1 [file sports-11-00026-s001.zip › sports-2110227 - supplementary.pdf]

Supplementary Materials

Table S1. Subjective load monitoring variables on *OwnUrGoal* across 10 weeks.

Variable	Week 1	Week 2	Week 3	Week 4	Week 5	Week 6	Week 7	Week 8	Week 9	Week 10
Wellness score	12.6 (4.8)	12.5 (4.8)	12.4 (4.8)	13.1 (4.8)	12.9 (4.5)	13.1 (4.8)	14.1 (4.4)	13.1 (4.3)	12.7 (4.7)	12.5 (4.8)
Muscle soreness	1.8 (0.9)	1.9 (0.8)	1.8 (0.9)	2.0 (1.1)	1.9 (1.0)	2.0 (1.0)	2.0 (0.9)	2.2 (1.0)	1.8 (0.9)	1.9 (1.0)
Fatigue	2.7 (1.3)	2.9 (1.2)	2.8 (1.4)	3.1 (1.3)	2.8 (1.4)	3.0 (1.4)	3.2 (1.4)	3.0 (1.1)	2.8 (1.2)	2.8 (1.3)
Sleep quality	2.6 (1.0)	2.6 (0.9)	2.6 (1.0)	2.9 (1.1)	2.7 (1.2)	2.8 (1.3)	3.0 (1.1)	2.9 (0.9)	2.7 (1.1)	2.6 (1.1)
Hours slept	8.7 (0.9)	8.6 (0.7)	8.7 (0.7)	8.5 (0.8)	8.6 (0.9)	8.7 (1.2)	8.5 (0.9)	8.5 (0.8)	8.6 (0.6)	**8.9**[cef] **(0.7)**
Stress	2.9 (1.2)	2.7 (1.3)	**2.6**[e] **(1.1)**	2.7 (1.1)	2.9 (1.3)	2.8 (1.2)	3.0 (1.2)	**2.6**[e] **(1.1)**	2.7 (1.1)	**2.5**[e] **(1.1)**
Motivation	2.6 (1.4)	2.5 (1.2)	2.6 (1.3)	2.5 (1.2)	2.7 (1.2)	2.6 (1.4)	2.7 (1.2)	2.5 (1.3)	2.7 (1.3)	2.6 (1.3)
Weekly training load	3612.5 (1637.5)	4300.6 (1089.9)	3971.1 (1754.5)	3716.1 (978.1)	**2689.4**[abcd] **(980.4)**	4051.6 (1003.9)	3587.7 (1119.3)	3741.4 (1754.3)	3315.6 (2563.4)	**2695.6**[abcd] **(1442.9)**

a < 0.05 vs Week 2; b < 0.05 vs Week 3; c < 0.05 vs Week 4; d < 0.05 vs Week 6; e < 0.05 vs Week 7; f < 0.05 vs Week 8.

Table S2. Objective load monitoring variables on *OwnUrGoal* across 10 weeks.

Variable	Week 1	Week 2	Week 3	Week 4	Week 5	Week 6	Week 7	Week 8	Week 9	Week 10
Session duration (mins)	97.9 (21.2)	104.6 (13.8)	111.2 (12.6)	**94.6**[bceh] **(7.1)**	107.3 (13.5)	**95.8**[c] **(16.9)**	107.3 (23.9)	107.1 (16.6)	97.4 (26.1)	**87.9**[bcegh] **(22.6)**
TRIMP.mod	133.0 (89.7)	160.8 (91.9)	**215.1**[af] **(53.6)**	154.3 (65.6)	**210.7**[af] **(86.8)**	131.2 (71.8)	**227.6**[af] **(111.6)**	**243.1**[af] **(88.8)**	**208.9**[af] **(74.9)**	**201.4**[af] **(87.8)**
TRIMP.mod/min	1.3 (0.7)	1.5 (0.7)	**1.9**[abf] **(0.4)**	1.6 (0.6)	**1.9**[af] **(0.7)**	1.4 (0.7)	**2.0**[abf] **(0.7)**	**2.3**[abdf] **(0.8)**	**2.2**[abdf] **(0.5)**	**2.3**[abdf] **(0.7)**
Max HR (bpm)	189.0 (7.0)	190.6 (7.9)	**197.6**[f] **(7.4)**	191.2 (9.0)	**192.8**[f] **(8.2)**	181.5 (13.4)	**199.1**[f] **(11.1)**	**196.1**[f] **(10.2)**	191.1 (13.0)	**194.7**[f] **(9.1)**
Avg. HR (bpm)	144.1 (10.1)	145.7 (8.4)	**155.3**[abdf] **(5.8)**	148.8 (6.9)	**153.6**[abf] **(9.8)**	140.7 (13.0)	**157.9**[abdf] **(14.8)**	**160.6**[abdf] **(11.6)**	**153.2**[abf] **(8.9)**	**158.7**[abdf] **(8.4)**
PL/Min. (AU)	79.0 (9.7)	**69.8**[achi] **(10.5)**	85.1 (12.9)	**77.4**[i] **(8.8)**	**76.5**[i] **(13.3)**	**74.1**[i] **(11.5)**	**77.9**[i] **(11.0)**	79.9 (10.0)	**91.3**[ah] **(11.2)**	**71.7**[achi] **(7.7)**

TRIMP.mod = Training impulse modified; HR = Heart rate; PL/Min. = PlayerLoad/minute; a < 0.05 vs Week 1; b < 0.05 vs Week 2; c < 0.05 vs Week 3; d < 0.05 vs Week 4; e < 0.05 vs Week 5; f < 0.05 vs Week 6; g < 0.05 vs Week 7; h < 0.05 vs Week 8; I < 0.05 vs Week 9.

Table S3. Countermovement jump outcome variables across 10 weeks.

Variable	Week 1	Week 2	Week 3	Week 4	Week 5	Week 6	Week 7	Week 8	Week 9	Week 10
JH (cm)	22.1 (3.3)	19.1 (3.9)	20.8 (3.6)	20.5 (3.6)	20.2 (4.8)	18.6 (3.9)	20.1 (3.7)	19.3 (4.6)	20.1 (4.7)	20.2 (3.6)
ToV (ms)	2.1 (0.2)	1.9 (0.2)	2.0 (0.2)	2.0 (0.2)	2.0 (0.2)	1.9 (0.2)	2.0 (0.2)	1.9 (0.2)	2.0 (0.2)	2.0 (0.2)
FT (s)	0.4 (< 0.1)	0.4 (< 0.1)	0.4 (< 0.1)	0.4 (< 0.1)	0.4 (0.1)	0.4 (< 0.1)	0.4 (< 0.1)	0.4 (< 0.1)	0.4 (< 0.1)	0.4 (< 0.1)
CT (s)	0.7 (0.2)	0.7 (0.1)	0.6 (0.1)	0.7 (0.1)	0.7 (0.1)	0.7 (0.1)	0.6 (0.1)	0.6 (0.1)	0.6 (0.1)	0.7 (0.1)
RSI.M	0.3 (0.1)	0.3 (0.1)	0.3 (0.1)	0.3 (0.1)	0.3 (0.1)	0.2 (0.1)	0.3 (< 0.1)	0.3 (0.1)	0.3 (0.1)	0.3 (0.1)
A.PF (N)	1709.7 (398.5)	1668.3 (322.8)	1776.2 (361.9)	1751.3 (285.3)	1701.5 (297.3)	1664.6 (328.9)	1733.3 (283.0)	1664.8 (327.4)	1721.7 (367.1)	1736.9 (326.4)
R.PF (N.kg.-1)	23.9 (3.1)	23.0 (2.3)	24.6 (2.5)	24.0 (2.3)	23.4 (2.9)	23.2 (1.9)	23.7 (2.1)	23.1 (2.4)	23.7 (3.0)	23.8 (2.6)
A.PLF (N)	2401.0 (501.3)	2402.2 (712.9)	2402.3 (705.7)	2449.8 (833.2)	2362.8 (654.1)	2127.4 (618.1)	2409.0 (761.8)	2375.1 (765.8)	2305.5 (734.0)	2381.6 (802.7)
R.PLF (N.kg.-1)	33.7 (4.0)	32.9 (6.6)	33.0 (5.6)	33.0 (7.5)	32.2 (6.8)	29.4 (4.1)	32.6 (7.8)	32.7 (7.2)	31.4 (6.9)	32.2 (7.2)
A.PP (W)	3213.9 (808.3)	3154.4 (791.5)	**3267.7[ab] (701.2)**	**3224.5[ab] (796.8)**	3142.5 (716.4)	3064.1 (778.2)	3177.4 (756.5)	3156.7 (729.1)	3174.8 (788.2)	**3225.2[ab] (820.5)**
R.PP (W.kg)	44.7 (4.7)	43.3 (5.3)	45.2 (3.8)	43.8 (5.7)	42.9 (5.4)	42.1 (4.7)	43.2 (5.1)	44.0 (8.5)	43.5 (6.6)	44.0 (5.9)
Ecc. AP (W)	-362.0 (106.0)	-392.8 (84.6)	-371.0 (87.9)	-392.8 (103.1)	-392.8 (97.4)	-359.4 (101.9)	-385.3 (71.7)	-388.4 (93.1)	-393.9 (101.9)	-387.7 (91.9)
Con. AP (W)	**1975.2[b] (484.6)**	1885.0 (422.0)	**2020.8[b] (423.8)**	**1937.7[b] (409.5)**	1853.2 (364.7)	1766.9 (333.0)	1906.8 (370.6)	1884.1 (402.6)	1878.2 (408.5)	**1907.8[b] (401.3)**

JH = jump height, ToV = take-off velocity; FT = flight time; CT = contact time; RSI.M = RSI Mod; A.PF = absolute peak force; R.PF = relative peak force; A.PLF = absolute peak landing force; R.PLF = relative peak landing force; A.PP = absolute peak power; R.PP = relative peak power; Ecc. AP = eccentric average power; Con. AP = concentric average power; a < 0.05 vs Week 5 ; b < 0.05 vs Week 6.

Table S4. Countermovement jump impulse variables across 10 weeks.

Variable	Week 1	Week 2	Week 3	Week 4	Week 5	Week 6	Week 7	Week 8	Week 9	Week 10
UW Imp. (Ns.kg-1)	-1.1 (0.2)	-1.1 (0.1)	-1.1 (0.2)	-1.1 (0.2)	-1.1 (0.3)	-1.1 (0.2)	-1.1 (0.2)	-1.1 (0.3)	-1.1 (0.2)	-1.2 (0.2)
STR Imp. (Ns.kg-1)	1.1 (0.2)	1.1 (0.1)	1.1 (0.2)	1.1 (0.2)	1.1 (0.2)	1.0 (0.2)	1.1 (0.2)	1.1 (0.3)	1.1 (0.2)	**1.2[b] (0.2)**
NI (Ns.kg-1)	2.4 (0.2)	2.4 (0.2)	2.4 (0.2)	2.3 (0.1)	2.4 (0.3)	2.4 (0.2)	**2.3[a] (0.2)**	2.4 (0.3)	**2.4[a] (0.2)**	**2.4[a] (0.2)**
PA-I Imp. (Ns.kg-1)	2.5 (0.2)	2.5 (0.2)	2.5 (0.2)	2.5 (0.2)	2.5 (0.3)	2.4 (0.2)	2.4 (0.2)	2.5 (0.3)	2.4 (0.2)	2.4 (0.2)
PA-II Imp. (Ns.kg-1)	0.1 (0.1)	0.1 (0.1)	0.1 (0.1)	0.1 (0.1)	0.1 (0.1)	0.1 (0.0)	0.1 (0.1)	0.1 (0.0)	0.1 (0.1)	0.1 (0.1)
PD Imp. (Ns.kg-1)	-0.4 (0.1)	-0.5 (0.1)	-0.4 (0.1)	-0.4 (0.1)	-0.4 (0.1)	-0.4 (0.1)	-0.4 (0.1)	-0.5 (0.2)	-0.4 (0.1)	-0.4 (0.1)
UW Dur. (ms)	382.4 (146.9)	316.7 (54.6)	324.9 (98.9)	313.9 (49.7)	324.9 (70.9)	334.9 (101.5)	304.3 (37.1)	325.8 (56.3)	307.9 (59.6)	328.9 (68.8)
STR Dur. (ms)	153.4 (35.7)	160.6 (31.0)	147.8 (34.0)	154.4 (40.5)	173.9 (46.7)	164.0 (34.9)	158.8 (36.4)	165.5 (30.9)	163.9 (41.9)	151.2 (38.0)
NI Dur. (ms)	206.9 (33.9)	219.6 (37.0)	197.4 (40.5)	201.2 (38.2)	222.9 (53.1)	212.8 (34.3)	204.7 (35.6)	216.4 (34.0)	215.5 (50.9)	209.8 (45.2)
PA-I Dur. (ms)	227.5 (38.2)	232.0 (35.4)	211.2 (31.7)	226.4 (42.9)	238.1 (51.5)	225.5 (31.5)	222.8 (37.5)	231.5 (32.1)	232.3 (48.3)	227.8 (38.9)
PA-II Dur. (ms)	20.5 (10.5)	12.4 (8.8)	13.8 (11.8)	16.9 (12.7)	15.2 (10.6)	12.7 (8.7)	18.2 (10.5)	15.2 (8.2)	16.9 (8.3)	18.0 (13.1)
PD Dur. (ms)	61.9 (8.9)	69.2 (6.7)	62.9 (7.7)	60.2 (7.3)	61.8 (6.1)	64.7 (5.7)	60.2 (5.9)	69.2 (13.8)	62.5 (8.2)	63.6 (9.9)

Imp. = impulse; Dur. = duration; UW = Unweighted phase; STR = Stretching phase; NI = Net impulse phase; PA-I = Propulsion acceleration I phase; PA-II = Propulsion acceleration II phase; PD = Propulsion deceleration; a < 0.05 vs Week 3; b < 0.05 vs Week 6.

Table S5. Isometric mid-thigh pull performance outcome variables across 10 weeks.

Variable	Week 1	Week 2	Week 3	Week 4	Week 5	Week 6	Week 7	Week 8	Week 9	Week 10
Mass	66.7	69.4	68.0	67.6	66.0	67.5	70.8	66.4	66.9	63.9
(kg)	(15.3)	(12.0)	(15.6)	(10.6)	(13.9)	(16.6)	(17.1)	(16.4)	(12.1)	(12.9)
A.PF	1634.9	1683.7	1616.3	1674.3	1633.2	1672.9	1628.4	1629.6	1617.6	1702.7
(N)	(281.0)	(224.4)	(248.7)	(271.7)	(323.0)	(295.1)	(274.2)	(295.8)	(297.3)	(262.0)
R.PF	25.2	24.6	24.3	25.0	25.4	25.7	25.6	25.7	24.6	27.1
(N.kg-1)	(4.6)	(2.8)	(3.3)	(3.8)	(5.9)	(6.6)	(4.1)	(6.9)	(4.9)	(4.6)
PF.D	2578.7	2476.8	2022.6	1843.6	2417.4	2695.0	2023.8	1800.5	2323.5	2279.3
(ms)	(1173.2)	(1291.2)	(1053.4)	(1359.3)	(1269.7)	(961.0)	(888.6)	(1127.0)	(1141.7)	(1173.2)
RFD.30	21879.2	11365.7	22149.8	21890.0	21276.1	21586.4	23025.0	21352.3	21298.5	20748.6
(N.s-1)	(5085.1)	(3863.2)	(5068.7)	(3661.1)	(4726.6)	(5505.4)	(5803.3)	(5423.2)	(4126.4)	(4276.8)
RFD.50	13110.5	13430.0	13294.4	13183.0	12766.2	12947.6	13803.3	12805.1	12786.6	12452.0
(N.s-1)	(3051.0)	(2301.6)	(3044.3)	(2250.0)	(2797.7)	3274.6)	(3497.5)	(3229.1)	(2467.1)	(2625.3)
RFD.90	7260.1	7489.3	7386.2	73626.5	7094.4	7197.1	7664.3	7129.0	7104.0	6918.5
(N.s-1)	(1688.9)	(1268.2)	(1686.1)	(1193.3)	(1518.5)	(1795.6)	(1964.2)	(1776.1)	(1368.9)	(1454.8)
RFD.100	6532.7	6750.9	6646.7	6624.8	6383.3	4679.1	6897.4	6422.9	6392.2	6226.8
(N.s-1)	(1520.2)	(1140.6)	(1515.6)	(1057.4)	(1358.7)	(1613.7)	(1770.4)	(1595.9)	(1231.2)	(1308.6)
RFD.150	4351.6	4546.1	4429.3	4407.5	4250.5	4323.1	4596.3	4294.9	4253.9	4150.7
(N.s-1)	(1018.5)	(764.8)	(764.8)	(1008.4)	(671.3)	(883.7)	(1067.2)	(1178.9)	(1059.7)	(813.0)
RFD.200	3254.8	3430.8	3321.9	3336.9	3186.9	3239.5	3455.8	3208.2	3189.2	3110.0
(N.s-1)	(765.8)	(585.6)	(760.92)	(558.6)	(677.7	(795.6)	(879.07)	(797.3)	(606.0)	(629.4)
RFD.250	2608.1	2742.6	2658.3	2964.0	**2552.0[ab]**	2593.7	2776.8	**2541.4[b]**	**2551.6[b]**	**2476.4[ab]**
(N.s-1)	(619.7)	(472.7)	(609.1)	(762.2)	**(560.9)**	(632.1)	(710.4)	**(642.9)**	**(491.2)**	**(496.9)**
pRFD	852.9	81.0	1134.2	2111.9	1041.8	694.5	1060.0	1347.1	1068.5	953.1
(N.s-1)	(669.4)	(481.4)	(890.1)	(2004.5)	(1059.6)	(264.8)	(767.4)	(1147.4)	(1008.3)	(417.5)
I.100	**0.2[b]**	**0.2[b]**	0.2	0.4	**0.2[b]**	**0.1[b]**	0.2	0.2	0.2	**0.2[b]**
(N.s-1)	**(0.1)**	**(0.1)**	(0.1)	(0.3)	**(0.1)**	**(0.1)**	(0.1)	(0.1)	(0.2)	**(0.1)**
I.250	1.0	1.0	1.0	1.6	1.0	1.0	1.1	1.2	1.1	1.1
(N.s-1)	(0.5)	(0.3)	(0.4)	(1.1)	(0.3)	(0.5)	(0.4)	(0.6)	(0.6)	(0.6)
PF.I	33.9	28.3	24.3	23.5	33.2	34.4	22.3	24.1	29.1	32.1
(N.s-1)	(20.7)	(15.1)	(15.0)	(18.7)	(27.3)	(17.1)	(11.4)	(17.3)	(15.6)	(25.6)

A.PF = absolute peak force; R.PF = relative peak force; PF.D = peak force duration; RFD.30 = rate of force development from onset to 30ms; RFD.50 = rate of force development from onset to 50ms; RFD.90 = rate of force development from onset to 90ms; RFD.100 = rate of force development from onset to 100ms; RFD.150 = rate of force development from onset to 150ms; RFD.200 = rate of force development from onset to 200ms; RFD.250 = rate of force development from onset to 250ms; pRFD = peak rate of force development; I.100 = impulse from onset to 100ms; I.250 = impulse from onset to 250ms; PF.I = peak force impulse. a < 0.05 vs Week 2; b < 0.05 vs Week 4

Table S6. Performance testing correlations.

		JH (cm)	A.PF (N)	PF.D (ms)
JH (cm)	r^2	1	.306*	.118
	Sig.		.001	.192
A.PF (N)	r^2		1	.223*
	Sig.			.012
PF.D (ms)	r^2			1
	Sig.			

JH = Jump height; A.PF = Absolute peak force; PF.D = Peak force duration; r^2 = Pearson Correlation; Sig. = Significance (2 tailed); * < 0.05.

Table S7. Correlations between athlete monitoring methods

		Wellness Score	WTL.Total	JH (cm)	A.PF (N)	TRIMP.Mod/Min	PL/Min. (AU)
Wellness Score	r^2	1	-.137	.049	.020	.222*	-.078
	Sig.		.107	.584	.822	.013	.379
WTL.Total	r^2		1	-.184*	-.236*	-.080	.025
	Sig.			.038	.008	.375	.780
JH (cm)	r^2			1	.306*	-.119	-.038
	Sig.				.001	.197	.676
A.PF (N)	r^2				1	-.169	-.280*
	Sig.					.065	.002
TRIMP.Mod/Min	r^2					1	.275*
	Sig.						.002
PL/Min. (AU)	r^2						1
	Sig.						

WTL.Total = Weekly training load total; JH = Jump height; A.PF = Absolute peak force; TRIMP.Mod/Min = Training impulse modified/minute; PL/Min = PlayerLoad/minute; r^2 = Pearson Correlation; Sig. = Significance (2 tailed); * < 0.05.

Table S8. Correlations between *OwnUrGoal* wellness variables and other athlete monitoring methods

		WTL.Total	JH (cm)	A.PF (N)	TRIMP.Mod/Min	PL/Min. (AU)
Muscle Soreness	r^2	-.153	.284*	.006	.020	.123
	Sig.	.072	.001	.947	.823	.167
Fatigue	r^2	-.026	-.032	-.016	.290*	-.010
	Sig.	.756	.724	.855	.001	.908
Sleep Quality	r^2	-.172*	-.084	.021	.201*	-.105
	Sig.	.043	.347	.816	.024	.236
Hours Slept	r^2	.094	.128	-.077	-.101	.101
	Sig.	.267	.152	.389	.262	.256
Stress	r^2	.014	-.033	-.040	.154	-.079
	Sig.	.874	.711	.659	.085	.373
Motivation	r^2	-.235*	.102	.118	.182*	-.207*
	Sig.	.005	.256	.186	.042	.019

WTL.Total = Weekly training load total; JH = Jump height; A.PF = Absolute peak force; TRIMP.Mod/Min = Training impulse modified/minute; PL/Min = PlayerLoad/minute; r^2 = Pearson Correlation; Sig. = Significance (2 tailed); * < 0.05.
